# Multiple sclerosis in Germany: data analysis of administrative prevalence and healthcare delivery in the statutory health system

**DOI:** 10.1186/1472-6963-14-381

**Published:** 2014-09-10

**Authors:** Ariane Höer, Guido Schiffhorst, Anne Zimmermann, Johann Fischaleck, Luise Gehrmann, Henrik Ahrens, Gunther Carl, Karl-Otto Sigel, Ulrike Osowski, Maria Klein, Hans-Holger Bleß

**Affiliations:** IGES Institute GmbH, Berlin, Germany; Bavarian Association of Statutory Health Insurance Physicians (BASHIP), München, Germany; German Association of Neurologists and Psychiatrists (BVDN), Krefeld, Germany; German Association of Neurologists (BDN), Krefeld, Germany; Merck Serono GmbH, Darmstadt, Germany; MSD Sharp & Dohme GmbH, Haar, Germany

## Abstract

**Background:**

Healthcare-utilization data for multiple sclerosis (MS) are scarce in Germany. The Purpose of the study was to analyse administrative prevalence of MS, medication use and type of specialists involved in MS treatment in the outpatient setting in Bavaria.

**Methods:**

Pseudonymized claims data from Bavarian Statutory Health Insurance (SHI)-accredited physicians were used. Administrative prevalence of MS was defined as having ≥1 MS diagnosis (International Classification of Diseases, 10th edition, code G35) documented by a neurologist or psychiatrist, or ≥1 prescription for disease-modifying drugs (DMDs)). The administrative prevalence calculated for Bavaria was projected to Germany. DMD prescription and involvement of different specialities in health care service for MS patients was analysed.

**Results:**

Administrative prevalence of MS in Bavaria increased from 0.123% to 0.175% of insured persons between 2005 and 2009; when projected, this yielded ~102,000–143,000 patients with MS in the German population. The percentage of patients receiving ≥1 DMD prescription increased from 45.5% to 50.5%. Patients with MS were mainly treated by neurologists in the ambulatory care setting.

**Conclusions:**

These results provide important information on the administrative prevalence of MS in Bavaria and on healthcare provision for patients, which is relevant for resource planning in the healthcare sector.

## Background

Multiple sclerosis (MS) is a chronic immune-mediated demyelinating disease of the central nervous system (CNS) and represents a major cause of neurological disability in young adulthood [1, 2]. The estimated prevalence of MS in Europe over the past three decades is 83 cases per 100,000, with higher rates in northern countries, and the mean annual incidence is 4.3 per 100,000 [3, 4].

In Germany, MS prevalence data vary between studies depending on several factors, including study population, region and methodology used. There are an estimated 100,000 to 140,000 patients with MS in Germany [5, 6], with an average age at diagnosis of 35 years, and diagnosis is 2.5 times more frequent in women than in men [7, 8]. In 80% of cases, the disease starts with a relapsing–remitting course, and some of these patients will develop secondary progressive MS (SPMS) [9, 10]. According to data from the German MS registry (2007), 55% of German patients suffer from relapsing–remitting MS (RRMS), 32% from SPMS and 9% from primary progressive MS (PPMS) [7].

The socio-economic impact of MS is high. In a 2006 German cost-of-illness analysis, the mean total annual cost of MS per patient was calculated as ~ €40,000, with costs increasing significantly with disease severity [8]. Two-fifths of the total cost are related to decreased productivity owing to sick leave and early retirement, while mean direct healthcare costs are €17,165, demonstrating that MS is a major healthcare burden in Germany [8]. Disease-modifying drugs (DMDs), especially interferon (IFN) beta, glatiramer acetate (GA) and natalizumab, account for the majority of direct costs. In addition, accompanying symptoms and conditions such as muscle spasms, fatigue, acute and chronic pain, depression or unstable bladder can require combination pharmacotherapy and neurorehabilitation.

In 2001, standardized MS diagnostic criteria based on clinical, laboratory and radiological findings (McDonald criteria) were first published [11]. These criteria and their subsequent revisions [12, 13] simplified the diagnostic process and contributed towards an earlier diagnosis of MS, in particular enabled by the use of magnetic resonance imaging (MRI) of the CNS, which allows detection of characteristic abnormalities that are found in >95% of patients [14]. Nonetheless, despite these improvements, the time to diagnosis is still ~3 years in Germany, suggesting that there are deficits in healthcare provision [7, 8].

The German MS registry [7], which was established in 2001, provides valuable information about patient characteristics and healthcare patterns in Germany. However, the data are not representative of the MS population in Germany owing to the fact that only a limited number of medical centres have participated in the registry and the proportion of rehabilitation centres with more severe cases of MS is high. In this study, we analysed insurance claims data from the outpatient care setting in Bavaria, Southern Germany, with the aim of providing further information on the administrative prevalence of MS, prescribed medications and medical specialties that are mainly involved in providing ambulatory healthcare for MS.

## Methods

### Data

Claims data from the Bavarian Association of Statutory Health Insurance Physicians (BASHIP) covering the period from 1 April 2005 to 31 December 2009 were used. These data covered about 10.4 m insured persons [15]. The total population of Bavaria counted 12.5 m people [16]. Thus, about 83% of the total Bavarian population were covered by the data. Available data were pseudonymized, and included patient age and sex, prescription details (active component, prescription month, specialty of the prescribing physician, dosage, number of packages and dosage forms) and details of ambulatory treatment cases (diagnoses according to the International Classification of Diseases, 10th edition [ICD-10], specialty of the attending physician and fee schedule item specification and number). The data covered all healthcare services delivered by BASHIP office-based physicians, and approximately 98% of drugs prescribed by BASHIP physicians reimbursed within the statutory health insurance (SHI) system.

The claims data have been collected for the purpose of reimbursing health care services and prescriptions by the SHI. The use of claims data beyond this purpose, e.g. for research, or to counsel SHI physicians is in accordance to § 300 of the German Social Security Code V (SGB V). This article rules the billing of drugs which have been dispensed by pharmacies and are reimbursed by the SHI. The pharmacies are allowed to task computing centers with the billing. On demand of the Associations of Statutory Health Insurance Physicians (*Kassenärztliche Vereinigung, KV*), the computing centers make available these claims data to the KV for the performance of their tasks according to § 73 article 8, § 84, and § 305 of SGB V. § 73 article 8 is related to the coverage of cost-effective prescribing, § 84 is related to the negotiations of drug budgets between the SHI and the KV, and § 305 says, that the KV counsels their SHI physicians concerning the delivered health services [17]. The claims data used for this analysis is not publicly available. Data analysis for the purpose of scientific investigations can be conducted in cooperation with the BASHIP [18].

### Selection of patients and administrative prevalence

Patients with MS were defined as having ≥1 diagnosis of MS (ICD-10 code G35) in the ambulatory care setting or ≥1 prescription for IFN beta-1a, IFN beta-1b, GA or natalizumab within the observation period. Only diagnoses by specialized physicians (Nervenärzte [i.e. multidisciplinary specialists in both neurology and psychiatry], neurologists and psychiatrists) were considered. For each patient and year, the first diagnosis (index diagnosis) was counted. Patients were identified based on their insurance number, which remains fixed during a period of insurance with a particular provider, but can change, for example in the case of marriage, retirement or health-insurance mergers. If patients received a new insurance number during the observation period, multiple counting may have occurred and cannot be eliminated because the required information is lacking.

### Drugs

The following DMDs were considered in detail: IFN beta-1a, IFN beta-1b, GA, mitoxantrone and natalizumab. Agents for symptomatic relief were grouped according to the German Association of Neurologists guidelines for the diagnosis and treatment of MS [9]: anti-dementia drugs, anti-depressants, anti-epileptics, selected muscle relaxants (baclofen, botulinum toxin, dantrolene, tizanidine, tolperisone, tetrazepam), urinary anti-spasmodics, selected medications to manage fatigue (amantadine, fampridine, modafinil), selected drugs for sexual dysfunction (sildenafil, tadalafil, tibolone) and selected drugs against tremor (propranolol).

### Data analysis

The administrative prevalence of MS was calculated based on the number of identified patients (differentiated by age group and sex) and the total Bavarian SHI population between 2005 and 2009. The prevalence was projected to the German SHI population and the general German population using statistical data from the Ministry of Health (number of SHI beneficiaries) [15] and from the Federal Statistical Office (total number of German residents) [16] for those years. From the BASHIP data, sex- and 5-year-age-group-specific prevalence was applied. Confidence intervals for administrative prevalence in the SHI population were calculated as Clopper-Pearson intervals. The Poisson distribution was used instead of the binomial distribution to derive cumulative probabilities, because the total number of patients in the SHI population was unknown.

For the analysis of drug and healthcare-service utilization, descriptive analyses were performed.

## Results

### Patient characteristics and prevalence of MS in Bavaria and Germany

During the observation period, 30,400 patients with MS were documented by office-based specialists of the Bavarian Association of Statutory Health Insurance Physicians (BASHIP). The number of patients identified annually ranged between 12,836 (2005) and 18,183 (2009). Owing to multiple counting in cases where the insurance number had changed or there was migration into or out of the Bavarian outpatient sector, the sum of the annual number of patients differed from the total number of patients.

The total administrative prevalence of MS in Bavaria (SHI population) increased from 0.123% in 2005 to 0.175% in 2009 (Table 1). The projection of this prevalence to the total German population resulted in estimated numbers of 101,702 and 142,856 patients with MS in 2005 and 2009, respectively. Of all identified patients in the Bavarian SHI population (n = 30,400), 73.1% (n = 22,226) were women (sex was unknown for 0.12% [n = 36] of patients), 44.0% (n = 13,387) were 30–44 years old, and 28.5% (n = 8,654) were 45–59 years old. The median (standard deviation [SD]) observation time was 1469 (567) days, and for 50.3% of patients the observation period lasted >4 years.

**Table 1:** **Administrative prevalence of MS**

Bavaria (SHI population)				Germany	
Year	Sex	Patients with MS	Administrative prevalence	Patients with MS, SHI population	Patients with MS, total population
		n1	% (95% CI)	n (95% CI)	n (95% CI)
2005	Men	3,361	0.069 (0.067; 0.072)	22,865(22,098; 23,651)	28,030(27,090; 28,994)
	Women	9,465	0.17(0.167; 0.174)	63,471(62,198; 64,762)	71,672(70,235; 73,131)
	All	12,826	0.123(0.121; 0.126)	86,589(85,097; 88,101)	101,702(99,949; 103,477)
2006	Men	3,773	0.078(0.076; 0.081)	25,634(24,823; 26,466)	31,450(30,455; 32,470)
	Women	10,579	0.191(0.187; 0.194)	70,810(69,467; 72,173)	80,049(78,531; 81,589)
	All	14,352	0.138(0.136; 0.14)	96,737(95,161; 98,333)	113,734(111,881; 115,611)
2007	Men	4,053	0.084(0.081; 0.086)	27,524(26,684; 28,385)	33,723(32,693; 34,778)
	Women	11,453	0.206(0.202; 0.21)	76,592(75,195; 78,008)	86,473(84,897; 88,072)
	All	15,506	0.149(0.147; 0.152)	104,444(102,807; 106,102)	122,636(120,713; 124,581)
2008	Men	4,494	0.093(0.09; 0.095)	30,419(29,536; 31,322)	37,189(36,109; 38,292)
	Women	12,381	0.223(0.219; 0.227)	82,585(81,136; 84,052)	93,130(91,497; 94,786)
	All	16,875	0.162(0.16; 0.164)	113,336(111,633; 115,059)	132,860(130,863; 134,880)
2009	Men	4,857	0.1(0.097; 0.103)	32,786(31,870; 33,721)	40,121(39,000; 41,265)
	Women	13,319	0.24(0.236; 0.244)	88,619(87,120; 90,137)	100,014(98,323; 101,728)
	All	18,176	0.175(0.172; 0.177)	121,754(119,991; 123,537)	142,856(140,786; 144,948)

Based on data from the SHI system in Bavaria and estimated administrative prevalence in Germany (SHI and total populations).

CI = confidence interval; MS = multiple sclerosis; SHI = Statutory Health Insurance.

1Patients of unknown sex were not considered for the estimation of prevalence. There were 10, 9, 5, 5 and 7 patients of unknown sex in 2005, 2006, 2007, 2008 and 2009, respectively, resulting in a total number of 12,836, 14,361, 15,511, 16,880 and 18,183 patients identified each year.

### Frequency and differentiation of MS diagnoses

**Figure 1:**
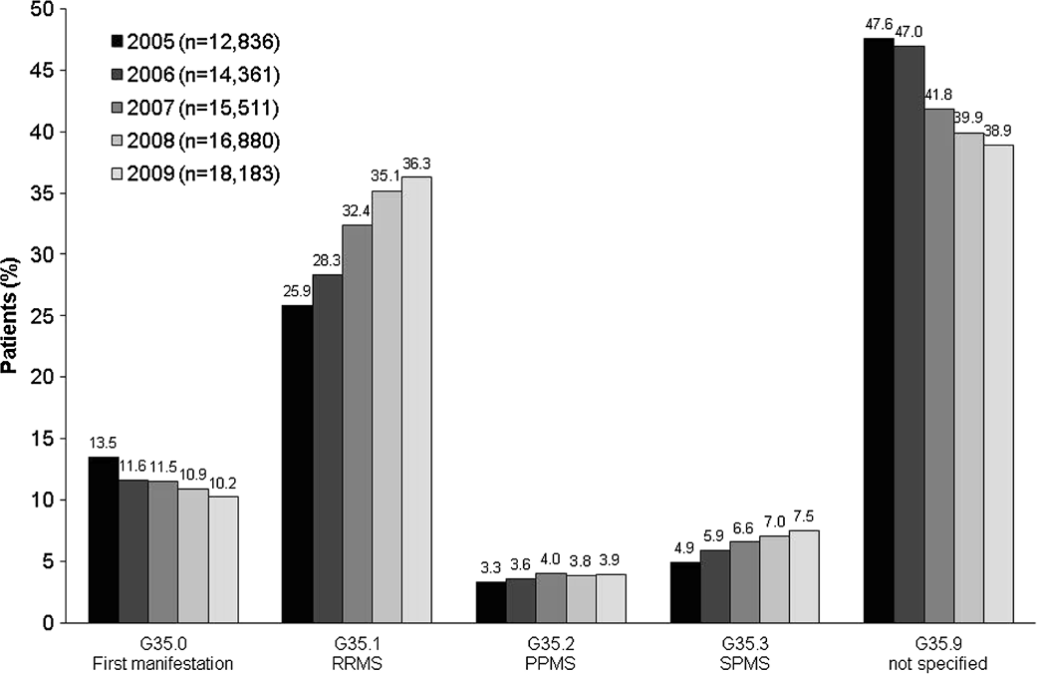
**Patient distribution according to multiple sclerosis index diagnosis for the years 2005–2009.** PPMS = primary progressive multiple sclerosis; RRMS = relapsing–remitting multiple sclerosis; SPMS = secondary progressive multiple sclerosis.

The German modification of ICD-10 comprises five MS diagnoses: G35.0 (first manifestation), G35.1 (RRMS), G35.2 (PPMS), G35.3 (SPMS) and G35.9 (not specified). For most patients, the index diagnosis was G35.9 (Figure 1); however, ICD-10 diagnoses increased from 25.9% in 2005 to 36.3% in 2009 for RRMS, and from 4.9% to 7.5% for SPMS. In 2009, RRMS was the most often documented diagnosis for patients aged <40 years (3978/9451 [42.1%]), whereas in patients aged ≥40 years, SPMS was the most frequent (3514/8732 [40.2%]). PPMS was mainly documented for patients ≥50 years of age (559/3512 patients [15.9%]). In each year, a small number of identified patients had no MS diagnosis recorded (2005: 620 [4.8%] patients; 2006: 513 [3.6%]; 2007: 574 [3.7%]; 2008: 542 [3.2%]; 2009: 579 [3.2%]).

### Prescription of DMDs

**Figure 2:**
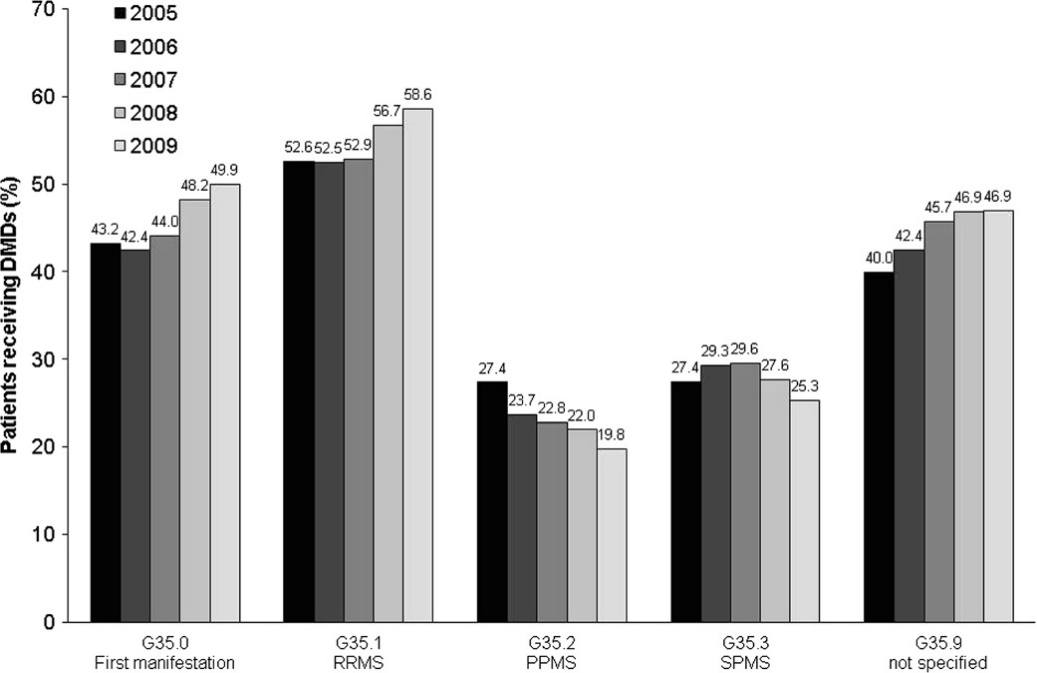
**Percentage of patients with prescriptions for DMDs, by multiple sclerosis index diagnosis during 2005–2009.** DMD, disease-modifying drug; PPMS = primary progressive multiple sclerosis; RRMS = relapsing–remitting multiple sclerosis; SPMS = secondary progressive multiple sclerosis.

The proportion of patients with ≥1 DMD prescription increased from 45.5% in 2005 to 50.5% in 2009. The highest percentage of prescriptions was found among patients with a diagnosis of RRMS, which rose from 52.6% to 58.6% (Figure 2). The proportion of patients with DMD prescriptions increased from 43.2% to 49.9% in patients with a first manifestation, and from 40.0% to 46.9% in those with a non-specified MS diagnosis. In the different age groups, the following increases in the proportions of patients with prescriptions were observed between 2005 and 2009: from 59.9% to 67.6% in patients <30 years of age, from 53.2% to 59.4% in patients aged 30–44 years, from 36.9% to 45.4% in patients aged 45–59 years, and from 17.2% to 18.5% in patients aged ≥60 years.

Most patients received prescriptions for IFN beta-1a and IFN beta-1b, although these rates decreased between 2005 and 2009, from 51.6% to 48.3% and from 22.4% to 19.9%, respectively. Conversely, the percentage of patients with GA prescriptions increased from 18.6% to 23.7%. Natalizumab, available in Germany since 2006, was prescribed for 7.0% of patients in 2009.

DMD prescriptions were mainly issued by specialized physicians. In 2009, 85.7% of IFN beta-1a prescriptions, 87.6% of IFN beta-1b prescriptions, 87.9% of GA prescriptions and 91.6% of natalizumab prescriptions were issued by Nervenärzte or neurologists.

### Drugs for symptomatic relief

The number of patients with prescriptions for symptomatic therapy increased from 4,938/12,836 (38.5%) in 2005 to 7,380/18,183 (40.6%) in 2009. In 2009, the most often prescribed drug was baclofen (9% of patients), followed by citalopram (8.0%), gabapentin (4.9%), tolperisone (4.2%) and mirtazapine (3.6%). Nervenärzte (36.0%) or neurologists (18.3%) filled most prescriptions. Prescriptions for NSAIDs were documented for 33.4% of patients and were mainly written by general practitioners (58.6%). Hypnotics and sedatives, opioids and neuroleptics were prescribed in 11.8%, 7.1% and 3.7% of cases, respectively. Prescriptions for corticosteroids for systemic use were recorded for 29.9% of patients and were mostly compiled by Nervenärzte (37.8%) or neurologists (28.7%).

### MRI of the neurocranium or spinal cord

The only diagnostic measure that is specific for MS and is specified by the fee schedule valid for German SHI physicians is MRI of the neurocranium or spinal cord (neuro-MRI). In 2005 (data for April to December only), 24.7% of patients underwent neuro-MRI scanning. After that, the percentage of patients for whom this diagnostic measure was used increased from 32.1% in 2006 to 33.6% in 2009, and was higher in patients receiving DMDs. In 2009, neuro-MRI scans were performed in 41.2% of patients with DMD prescriptions versus 29.2% of those without prescriptions. Similar differences were seen for all other years, including 2005 (data not shown).

## Discussion

The main objective of this analysis was to provide information on the epidemiology of MS and healthcare utilization in patients with MS in Germany, using claims data from SHI-accredited physicians in Bavaria.

Patient characteristics were consistent with previous publications [1, 7]: women were almost three times more likely than men to have MS, and the most affected age group was 30 to <60 years, suggesting that MS mainly concerns the working-age population.

In Bavaria, the number of patients with MS increased by 41.7% between 2005 and 2009. Projected patient numbers calculated for Germany based on these data (101,702 in 2005 and 142,856 in 2009) fit with observations from other German epidemiological studies that report a range of 100,000–140,000 patients with MS [5, 6]. The exact reasons for the increase in MS administrative prevalence are not known, but it should be taken into account that there were major revisions of the MS diagnostic criteria during the observation period [19], which may have allowed an earlier diagnosis (at the time of the first demyelinating event or clinically isolated syndrome [CIS]) and simplified the diagnostic process through the use of MRI. In addition, the capacity to perform MRI scans has increased substantially in Germany over the last 10 years, which, combined with the revisions in diagnostic criteria, could have led to an increase in the number of diagnoses made. Although the current analysis is not directly comparable with population-based epidemiological studies, the results are consistent with a meta-analysis that showed that the prevalence and incidence of MS generally increased over the last three to six decades, especially in women [20]. It is not possible to indicate the extent to which the observed increase in administrative MS prevalence is due to an actual increase.

There was a difference in the number of patients who were identified annually compared to the number of MS patients counted during the total observation period. The difference between the annual count and the total count is due to different factors, e.g. migration between the German states. But the main reason is double counting because the insurance number of the patient – by which the patients are identified – changes (for reasons see Methods). It is not possible to specify the exact proportion of patients, who have been counted twice or more. There is also no information, if there may be differences in the proportion of double counting considering morbidity, age, or sex. A crude estimation of the proportion of patients can be made considering the difference between the annual patient numbers and the total numbers as well as the number of years. This estimation indicates about 13% of the patients with possible double counting. Taking this possible double counting in consideration, the administrative MS prevalence calculated from the data of this analysis is probably overestimated. It can be expected that the true prevalence is about 10% lower.

As the majority of patients had a non-specified diagnosis of MS (ICD-10 code G35.9), our data provide only partial information on the frequency of the different MS subtypes in Bavaria. However, the results are consistent with existing studies showing that the majority of MS cases begin with a relapsing–remitting course, which may evolve into a secondary progressive course. If untreated, ~50% of patients develop SPMS within 10 years [9, 10]. The decrease in use of the diagnosis codes G.35.0 (first manifestation) and G35.9 (not specified), and increase in use of the more specific diagnosis codes (G35.1, G35.2 or G35.3), suggest that new patients are more often diagnosed with specific codes.

The percentage of patients who received ≥1 DMD prescription increased over the years (45.5% in 2005 to 50.5% in 2009). IFN beta-1a and IFN beta-1b were the most frequently prescribed drugs, accounting for over two-thirds of prescriptions, while prescriptions for GA increased to almost one-quarter of the total by 2009. These results are consistent with a similar analysis of claims data from the largest German SHI (8.8 million beneficiaries) that reported an increase in DMD prescriptions in the German MS population from 33.3% in 2004 to 40.9% in 2008, the majority being prescriptions for IFN beta [21]. These percentages are lower compared with data from the German MS registry, which reported that 71% of patients with MS received DMDs during 2005–2006; however, a high proportion of patients covered by the MS registry are treated in rehabilitation and specialized medical centres and require drug treatment [7].

An increase in DMD prescriptions was also observed in a Canadian study that had analysed population data from International Medical Statistics Health for 2002–2007 and reported a rise in DMD prescriptions by approximately 30% [22]. The increase observed in Germany followed a revision of the MS guidelines in 2006 that introduced major changes [19], such as recommendations for early DMD treatment at the time of the first demyelinating event or CIS, use of MRI to facilitate diagnosis and early treatment, and prolonged treatment with DMDs based on long-term efficacy and safety data for IFN beta.

Our analysis found that the percentage of patients with relevant MRI scans (neurocranium or spinal cord) was substantially higher in patients treated with DMDs; this is probably due to the fact that MRI scans are used in these patients to assess whether brain and spinal cord lesions are reduced by DMD use. Finally, the study showed that Nervenärzte and neurologists are the specialists most involved in the medical treatment of patients with MS within the office-based sector, which is in accordance with MS guidelines stating that the neurologist is the responsible physician for differential diagnosis, treatment initiation and follow-up.

This study is subject to certain limitations. The information available for the diagnosis of MS did not include any clinical measures such as MRI scans or laboratory parameters; therefore data quality relied solely on the diagnosis and prescriptions made by physicians. The criteria on which physicians based their decisions remain unknown. The data base includes data from all members of the SHI in Bavaria, which means that 83% of the total Bavarian population are covered (see Methods). Therefore, the data reflect the MS prevalence and health care referring to the great majority of the Bavarian population, although it cannot be excluded that there are some differences compared to the part of the population without access to the health care services covered by the SHI. Furthermore, the SHI population of Bavaria may differ from the overall German population in terms of demographic characteristics and/or morbidity. For this reason, age-group- and sex-specific prevalence was used for the projection of the numbers onto the German population. It is also possible that physicians in Bavaria differ in their prescribing and diagnostic behaviour owing to different incentives set by the regional association of SHI-accredited physicians for reporting medical procedures. Finally, the data cover only patients who had already received a diagnosis or treatment for MS. Patients without a diagnosis or MS-specific treatment (e.g. patients with very inactive MS who have not received medical attention or treatment during the time period surveyed) were not included, possibly underestimating the prevalence of MS. On the other hand, it was not possible to identify cases in which the initial diagnoses of MS were incorrect. This may result in a slight overestimation of the prevalence of MS.

## Conclusions

Our study shows that the administrative prevalence of MS in Germany has increased in recent years. Owing to the availability of new oral drugs that will lead to an increase in the number of treated patients, a further rise in administrative prevalence can be expected in the future. A growing proportion of patients receive DMDs, in particular IFN beta, although the use of GA is increasing. Ambulatory healthcare services for patients with MS are delivered mainly by specialized physicians.
